# Novel Molecular Approaches in Heart Failure: Seven Trans-Membrane Receptors Signaling in the Heart and Circulating Blood Leukocytes

**DOI:** 10.3389/fcvm.2015.00013

**Published:** 2015-03-16

**Authors:** Gabriele Giacomo Schiattarella, Fabio Magliulo, Fabio Cattaneo, Giuseppe Gargiulo, Anna Sannino, Anna Franzone, Marco Oliveti, Cinzia Perrino, Bruno Trimarco, Giovanni Esposito

**Affiliations:** ^1^Department of Advanced Biomedical Sciences, Federico II University, Naples, Italy; ^2^Department of Molecular Medicine and Medical Biotechnology, Federico II University, Naples, Italy; ^3^Department of Cardiology, Swiss Cardiovascular Center Bern, Bern, Switzerland

**Keywords:** cardiac remodeling, seven trans-membrane receptors, leukocytes, heart failure

## Abstract

Heart failure (HF) is the result of molecular, cellular, and structural changes induced by cardiac load or injury. A complex network of signaling pathways have been involved in the development and progression of cardiac dysfunction. In this review, we summarize the pivotal role of seven trans-membrane receptors (7TMRs), also called G-protein-coupled receptors (GPCRs), in HF. Moreover, we will discuss the current knowledge on the potential mirroring of 7TMR signaling between circulating blood leukocytes and the heart, and the related future possibilities in the management of HF patients.

## Introduction

Heart failure (HF) is the final end result of virtually all forms of cardiac disease, and a major cause of morbidity and mortality worldwide (1). In response to myocardial damage or enhanced workload, the heart undergoes a progressive anatomical and functional transformation, currently known as “remodeling” (2). Molecular, cellular, and interstitial changes contribute to determine such changes in size, shape, and function of the heart (3). While in an initial phase, these adaptations might enable the heart to maintain an almost normal function despite the injury, progressive remodeling is usually deleterious, and associated with a poor prognosis (4). Indeed, the inhibition of cardiac remodeling in animal models has been consistently proven to improve ventricular function (5). Therefore, the identification of the crucial signaling pathways involved in the morphological and functional modifications associated with pathological cardiac remodeling might be extremely important to prevent the development of HF.

A vast amount of data demonstrates that the superfamily of cell surface receptors seven trans-membrane receptors (7TMRs), also called G protein-coupled receptors (GPCRs) are the most important regulators of several cardiac functions, including heart rate, contractility, and remodeling (6). 7TMR signaling is tightly regulated, and prolonged agonist binding to the receptor promotes rapid receptor phosphorylation by GPCR kinases (GRKs). GRK2 is the most abundant isoform in the heart, and the best investigated (6). GRK2 expression and activity are markedly elevated in pathological cardiac remodeling and failure, and play a central role in the development and progression of cardiac dysfunction (7). GRK2-mediated 7TMR phosphorylation invariably uncouples the receptor from its signal-transducing G protein, and enhances its affinity for a family of cytosolic proteins known as β-arrestins (8). β-arrestins binding uncouples 7TMRs from G proteins, and promotes subsequent receptor internalization and eventually degradation. In addition to receptor desensitization, it has been recently recognized that β-arrestins and GRKs (specifically GRKs 5 and 6) participate also in further signal propagation in a G protein-independent fashion (9), by assembling macromolecular complexes, activating different signal transduction pathways, and regulating other receptor families, such as tyrosine kinase receptors and serine/threonine receptors.

Excessive neuro-hormonal activation associated with cardiac injury or overload induces extensive 7TMR signaling perturbations, and the modulation of 7TMR signaling in several different animal models of cardiac overload has been consistently shown to ameliorate cardiac remodeling and function (10). Thus, many efforts have been made to identify the crucial pathways involved in pathological cardiac remodeling and reliable circulating markers of molecular abnormalities occurring in the heart. The conceptual basis for these investigations is that cardiovascular diseases, and particularly HF, are systemic disorders in which a complex interplay between different organs occurs.

## Crosstalk between Circulating Blood Leukocytes and the Heart

**Figure 1:**
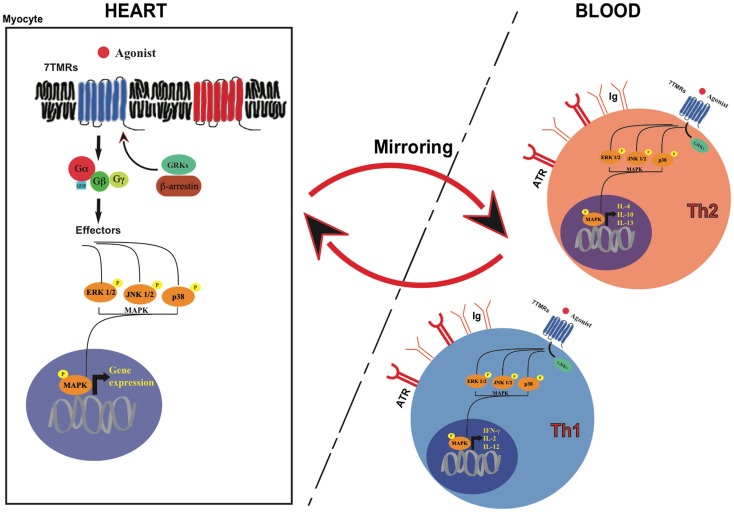
**The “paradigm” of lymphocyte mirroring of heart signals**.

HF is a multi-organ disorder originating in the heart and affecting many other extra-cardiac sites, including the immune system (11, 12). Inflammation plays a key role in the progressive deterioration of cardiac function by inducing ventricular dilatation, contractile dysfunction, fibrosis, and both apoptotic and necrotic cardiomyocyte death (13, 14). Immune system activation and neuro-hormonal perturbations are two strictly correlated processes, amplifying each other’s effects in a cascade (12, 15). It has been clearly recognized that autonomic nervous system perturbations, a hallmark of HF, determine the activation of the immune system. β-adrenergic receptors are expressed in lymphocytes and monocytes, and sympathetic stimulation has an activating effect on these cells inducing cytokines expression and release (16, 17). Autonomic nervous system deregulation is also characterized by increased production and secretion of angiotensinogen, which, through its conversion into angiotensin-II (ANG-II) and the subsequent induction of aldosterone production, promotes oxidative stress, inflammatory state, and cytokine expression both in the myocardium and circulating leukocytes (18). As a consequence, it has been proposed that abnormalities of 7TMR signaling (in particular, adrenergic signaling) in peripheral leukocytes might mirror those occurring in the heart, and particularly the molecular modifications of patients with pathological remodeling or overt HF (13, 19). In particular, *in vivo* studies have shown, in the past, that alterations of β-adrenergic receptors (βARs) system or the activation of MAPKs in white blood cells can mirror the modifications that are present in the heart (14) (Figure 1).

Immune activation leads to the recruitment of different populations of white blood cells, participating to various phases of cardiac remodeling. It is becoming increasingly clear that specific cell populations might exert specific roles in these processes. In particular, it has been suggested that neutrophils might play a significant role in the early response to myocardial ischemia, since innate immune signals rapidly recall these cells to clear the infarct area from dead cells and matrix debris, and to activate fibroblasts and matrix metalloproteinases (20). In contrast, monocyte/macrophage cells seem to be the leading actors of the second phase of post-infarct myocardial remodeling, persisting for many days in the infarct area and contributing to healing and scar formation by phagocytosis, neo-angiogenesis, and collagen deposition (21). During post-ischemic cardiac remodeling, dendritic cells are also mobilized from spleen in the systemic circulation and might exert a critical function, albeit still poorly studied, in modulating immune system activation (22).

7TMR signaling has been extensively investigated in lymphocytes, since they represent a relatively uniform population of cells capable of similar receptor-mediated functions. Several lines of evidence have shown that T lymphocytes exert an important role in cardiovascular remodeling and heart failure (23, 24). T-helper lymphocytes responses can be classified into T-lymphocyte helper type 1 (Th1) and type 2 (Th2) according to the predominant cytokines involved. Th1 responses include secretion of the cytokines IL-2, IL-12, and IFN-γ. Th2 response is characterized by IL-4, IL-10, and IL-13 production. Th1/Th2 imbalance, i.e., a disequilibrium in T-helper responses polarized to Th1 cell activation, has been described in many autoimmune diseases, and recent observations suggest that it might also be involved in coronary artery disease and in the progression toward heart failure (25, 26). Th1 response and its associated cytokine production, firstly interferon γ (INF-γ), have been associated with cardiac hypertrophy, increased interstitial fibrosis and cardiac dysfunction (27). Interestingly, some of the classic drugs used for the treatment of cardiovascular diseases and HF appear to equilibrate this imbalance in favor of Th2 responses (26, 28).

Interestingly, lymphocytes are characterized by a 7TMR expression pattern very similar to cardiomyocytes, endothelial cells, and vascular smooth muscle cells (VSMCs), and in particular α-adrenergic receptors (α-ARs), β-adrenergic receptors (βARs), and ANG-II receptors are well expressed (29–31). Previous, historical studies have analyzed β_2_AR density and responsiveness in lymphocytes from patients affected by arterial hypertension. After an initial increase in βAR density and responsiveness in the first phases of hypertension (32), desensitization of βARs has been observed (33). Interestingly, this phenomenon seems reversible, since normalization of sodium salt dietary intake partially restored the impairment in cyclic-AMP production after isoproterenol administration to cultured lymphocytes from hypertensive subjects (33).

Pressure and volume overload triggers a sustained down-regulation of βARs in lymphocyte plasma membranes, which has been demonstrated to correlate with βARs density in the myocardium of patients with heart valve disease (14). A similar correlation has been also described in patients with HF (13), wherein the hyperadrenergic state determines cardiac and lymphocyte βAR dysfunction, partially reversible after pharmacological inhibition of sympathetic overstimulation or with an improvement of the hemodynamic conditions (13). Interestingly, beta-blocker therapy has been shown to reduce Th1 polarization in CD4+ T-helper cells, leading to a significant decrease in the generation of IFN-γ (34). Moreover, in patients with HF, chronic therapy with beta-blockers and angiotensin converting hormone inhibitors has been shown to decrease 7TMR activation in peripheral CD4+ T-helper lymphocytes, to ameliorate the TH1/TH2 ratio, and to exert a beneficial effect on the immune system (35). This beta-blocker induced shift toward TH2 polarization has been associated with increased cAMP levels within peripheral T-helper lymphocytes from patients with HF (28). According to this view, T-helper cells might really represent a new potential target for pharmacological modulatory strategies in patients with HF. These insights might offer novel additional tools in the future management of HF patients.

Lymphocytes mirroring of 7TMR signaling in cardiac tissues might also involve other downstream 7TMR molecular targets, such as GRKs (36) or mitogen-activated protein kinases (MAPK) (Figure 1). GRK2 levels and activation have been shown to directly correlate to the amount of sympathetic outflow and inversely correlate to sensitivity and responsiveness to adrenergic signals, both in hypertension and HF (36, 37). A significant increase in GRK2 levels, already demonstrated in failing hearts, has been observed also in lymphocytes from HF patients: molecular studies on paired failing heart biopsies and circulating lymphocytes from the same patients have shown a significant inverse correlation between GRK2 activity and βARs responsiveness (37). Recently, a correlation between increased GRK2 levels and vascular dysfunction has been also demonstrated in lymphocytes (38). In this study, hypertensive patients were characterized by impaired vasodilatation after isoprenaline injection when compared with normotensive subjects, with a partial restore after the injection of the non-specific GRKs inhibitor heparin (38). These data suggest that hypertension and pressure overload induce a hyperadrenergic state that affects similarly cardiac and peripheral βARs signaling.

7TMR dysregulation is a hallmark of HF, and some of most effective pharmacological therapies in these patients, including βAR-blockers, have been shown to ameliorate βAR signaling (39). Interestingly, the administration of the beta-adrenergic blocker metoprolol has been shown to reduce GRK2 expression in peripheral blood lymphocytes from advanced elderly patients with HF (40, 41). Moreover, mechanical therapy with left ventricular assist devices, which represents a recent chance for the treatment of refractory, end stage HF as a bridge to heart transplant or as a destination therapy for patients who do not meet criteria for heart transplant, has been also shown to restore βAR function at multiple levels (39). Indeed, in these patients, a restoration of myocardial beta-adrenergic receptor signaling, assessed by membrane beta-adrenergic receptor density, adenylyl cyclase activity, and GRK2 expression and activity, has been observed after implantation of the assist device (42). Hata and coworkers have also shown that cardiac reduction of GRK2 activation after left ventricular assist device is mirrored by peripheral lymphocytes (43). More recently, Akter and coworkers have correlated the decreased levels of activation of GRK2 in peripheral lymphocytes of patients subjected to left ventricular assist device with an increased total beta-adrenergic receptor density on plasma membrane, and an augmented basal and isoproterenol-induced cyclic-AMP production in the myocardium (44). In a recently published manuscript, Rengo and coworkers have observed a significant reduction in lymphocyte GRK2 protein levels in 193 HF patients after physical exercise, obtained by a 3-month program of training (45). Not surprisingly, HF patients who did not show reduced lymphocyte GRK2 protein levels after training had a worst outcome (45).

We have recently analyzed the correlation between cardiac pressure overload and the activation of mitogen-activated protein kinases (MAPKs), extracellular-signal regulated kinase (ERK), c-Jun terminal kinase (JNK), and p38 in myocardial tissues or peripheral blood leukocytes from mice undergoing transverse aortic constriction (46). Cardiac activation of ERK, JNK, and p38 was significantly increased by pressure overload, and correlated with a consistent and coherent activation of the same MAPKs in leukocytes from the same animals (46). Furthermore, ERK phosphorylation was increased in leukocytes isolated from hypertensive patients with uncontrolled values of arterial blood pressure compared to normotensive volunteers, while leukocytes isolated from patients with controlled blood pressure displayed reduced MAPK activation. These results suggest that MAPKs might be sensors of cardiac pressure overload, and suggest that leukocytes might represent important cellular targets mirroring cardiac signaling (46).

It is still unclear, however, whether all these observations concerning lymphocytes represent only a passive phenomenon and a surrogate of cardiac remodeling processes, or “active” modifications with a specific pathophysiological role. It is worth to report that a similar mirroring phenomenon in peripheral lymphocytes has been described for the endocannabinoid system in an interesting number of diseases with a neuro-inflammatory basis, such as Huntington’s disease, Parkinson’s disease, multiple sclerosis, attention-deficit/hyperactivity disorder, schizophrenia, depression, and headache (47). Notably, similar modifications are poorly described in other classes of white blood cells.

## Conclusive Remarks

Despite several numbers of studies, a great deal of characterization is still required to fully understand the mechanisms involved in HF. Obviously, a huge limitation for basic research in HF is related to the difficulty in collecting human myocardial specimens for *in vivo* analysis. Such limitations have primarily raised the interest on circulating “mirrors” of cardiomyocytes. Thus, the phenomenon of mirroring in peripheral lymphocytes might represent an exciting and useful tool to non-invasively assess and monitor signal abnormalities in HF, with a feasible relevance for diagnosis, prognostic assessment, and therapy. At the same time, this concept should not be extremely forced to the assumption that every signal modifications in the heart might always be reproduced in peripheral lymphocytes. Although results from these studies are very promising and exciting, further investigations will be needed in the future to better understand the true biological meaning of mirroring and to define specific cell populations and new candidate signaling pathways.

## Conflict of Interest Statement

The authors declare that the research was conducted in the absence of any commercial or financial relationships that could be construed as a potential conflict of interest.

## References

[1] Liu L Eisen HJ. Epidemiology of heart failure and scope of the problem. Cardiol Clin (2014) 32(1):1–8,vii. 10.1016/j.ccl.2013.09.009 24286574

[2] Vilahur G Juan-Babot O Pena E Onate B Casani L Badimon L. Molecular and cellular mechanisms involved in cardiac remodeling after acute myocardial infarction. J Mol Cell Cardiol (2011) 50(3):522–33. 10.1016/j.yjmcc.2010.12.021 21219908

[3] Cohn JN Ferrari R Sharpe N. Cardiac remodeling – concepts and clinical implications: a consensus paper from an international forum on cardiac remodeling. Behalf of an international forum on cardiac remodeling. J Am Coll Cardiol (2000) 35(3):569–82. 10.1016/S0735-1097(99)00630-0 10716457

[4] Gaudron P Eilles C Kugler I Ertl G. Progressive left ventricular dysfunction and remodeling after myocardial infarction. Potential mechanisms and early predictors. Circulation (1993) 87(3):755–63. 10.1161/01.CIR.87.3.755 8443896

[5] Esposito G Rapacciuolo A Naga Prasad SV Takaoka H Thomas SA Koch WJ Genetic alterations that inhibit in vivo pressure-overload hypertrophy prevent cardiac dysfunction despite increased wall stress. Circulation (2002) 105(1):85–92. 10.1161/hc0102.101365 11772881

[6] Rockman HA Koch WJ Lefkowitz RJ Seven-transmembrane-spanning receptors and heart function. Nature (2002) 415(6868):206–12. 10.1038/415206a 11805844

[7] Cattaneo F Guerra G Parisi M De Marinis M Tafuri D Cinelli M Cell-surface receptors transactivation mediated by G protein-coupled receptors. Int J Mol Sci (2014) 15(11):19700–28. 10.3390/ijms151119700 25356505 PMC4264134

[8] Gurevich VV Gurevich EV. The structural basis of arrestin-mediated regulation of G-protein-coupled receptors. Pharmacol Ther (2006) 110(3):465–502. 10.1016/j.pharmthera.2005.09.008 16460808 PMC2562282

[9] Shenoy SK McDonald PH Kohout TA Lefkowitz RJ. Regulation of receptor fate by ubiquitination of activated beta 2-adrenergic receptor and beta-arrestin. Science (2001) 294(5545):1307–13. 10.1126/science.1063866 11588219

[10] Perrino C Rockman HA. Reversal of cardiac remodeling by modulation of adrenergic receptors: a new frontier in heart failure. Curr Opin Cardiol (2007) 22(5):443–9. 10.1097/HCO.0b013e3282294d72 17762546

[11] Yndestad A Damas JK Oie E Ueland T Gullestad L Aukrust P. Systemic inflammation in heart failure – the whys and wherefores. Heart Fail Rev (2006) 11(1):83–92. 10.1007/s10741-006-9196-2 16819581

[12] Jankowska EA Ponikowski P Piepoli MF Banasiak W Anker SD Poole-Wilson PA. Autonomic imbalance and immune activation in chronic heart failure – pathophysiological links. Cardiovasc Res (2006) 70(3):434–45. 10.1016/j.cardiores.2006.01.013 16480965

[13] Yamada S Ohkura T Uchida S Inabe K Iwatani Y Kimura R A sustained increase in beta-adrenoceptors during long-term therapy with metoprolol and bisoprolol in patients with heart failure from idiopathic dilated cardiomyopathy. Life Sci (1996) 58(20):1737–44. 10.1016/0024-3205(96)00155-5 8637398

[14] Dzimiri N Basco C Moorji A Afrane B Al-Halees Z. Characterization of lymphocyte beta 2-adrenoceptor signalling in patients with left ventricular volume overload disease. Clin Exp Pharmacol Physiol (2002) 29(3):181–8. 10.1046/j.1440-1681.2002.03625.x 11906480

[15] Mann DL. Stress-activated cytokines and the heart: from adaptation to maladaptation. Annu Rev Physiol (2003) 65:81–101. 10.1146/annurev.physiol.65.092101.142249 12500970

[16] Werner C Werdan K Ponicke K Brodde OE. Impaired beta-adrenergic control of immune function in patients with chronic heart failure: reversal by beta1-blocker treatment. Basic Res Cardiol (2001) 96(3):290–8. 10.1007/s003950170060 11403423

[17] Kohm AP Sanders VM. Norepinephrine and beta 2-adrenergic receptor stimulation regulate CD4+ T and B lymphocyte function in vitro and in vivo. Pharmacol Rev (2001) 53(4):487–525. 11734616

[18] Ahokas RA Warrington KJ Gerling IC Sun Y Wodi LA Herring PA Aldosteronism and peripheral blood mononuclear cell activation: a neuroendocrine-immune interface. Circ Res (2003) 93(10):e124–35. 10.1161/01.RES.0000102404.81461.25 14576195 PMC2896314

[19] Colucci WS Alexander RW Williams GH Rude RE Holman BL Konstam MA Decreased lymphocyte beta-adrenergic-receptor density in patients with heart failure and tolerance to the beta-adrenergic agonist pirbuterol. N Engl J Med (1981) 305(4):185–90. 10.1056/NEJM198107233050402 6113543

[20] Frangogiannis NG. The immune system and the remodeling infarcted heart: cell biological insights and therapeutic opportunities. J Cardiovasc Pharmacol (2014) 63(3):185–95. 10.1097/FJC.0000000000000003 24072174 PMC3949163

[21] Nahrendorf M Swirski FK Aikawa E Stangenberg L Wurdinger T Figueiredo JL The healing myocardium sequentially mobilizes two monocyte subsets with divergent and complementary functions. J Exp Med (2007) 204(12):3037–47. 10.1084/jem.20070885 18025128 PMC2118517

[22] Anzai A Anzai T Nagai S Maekawa Y Naito K Kaneko H Regulatory role of dendritic cells in postinfarction healing and left ventricular remodeling. Circulation (2012) 125(10):1234–45. 10.1161/CIRCULATIONAHA.111.052126 22308302

[23] Yu Q Watson RR Marchalonis JJ Larson DF. A role for T lymphocytes in mediating cardiac diastolic function. Am J Physiol Heart Circ Physiol (2005) 289(2):H643–51. 10.1152/ajpheart.00073.2005 16014617

[24] Yndestad A Ueland T Oie E Florholmen G Halvorsen B Attramadal H Elevated levels of activin A in heart failure: potential role in myocardial remodeling. Circulation (2004) 109(11):1379–85. 10.1161/01.CIR.0000120704.97934.41 14993131

[25] Cheng X Liao YH Ge H Li B Zhang J Yuan J TH1/TH2 functional imbalance after acute myocardial infarction: coronary arterial inflammation or myocardial inflammation. J Clin Immunol (2005) 25(3):246–53. 10.1007/s10875-005-4088-0 15981090

[26] Cheng X Ding Y Xia C Tang T Yu X Xie J Atorvastatin modulates Th1/Th2 response in patients with chronic heart failure. J Card Fail (2009) 15(2):158–62. 10.1016/j.cardfail.2008.10.001 19254676

[27] Levick SP Goldspink PH. Could interferon-gamma be a therapeutic target for treating heart failure? Heart Fail Rev (2014) 19(2):227–36. 10.1007/s10741-013-9393-8 23589353 PMC3844057

[28] Tian X Zhang L Hou Y Xu W Dong Y Liu J Effects of cAMP and beta-adrenergic receptor antagonists on the function of peripheral T helper lymphocytes in patients with heart failure. Neuroimmunomodulation (2011) 18(2):73–8. 10.1159/000319375 20938210

[29] Feldman RD Limbird LE Nadeau J Robertson D Wood AJ. Leukocyte beta-receptor alterations in hypertensive subjects. J Clin Invest (1984) 73(3):648–53. 10.1172/JCI111255 6323524 PMC425064

[30] Peng Y Ma S Zhang S Li Y Yang L Bian S. Clinical significance of changes in beta-adrenoreceptors in peripheral lymphocytes in patients with essential hypertension. Chin Med J (Engl) (2000) 113(12):1064–7. 11776136

[31] Ricci A Bronzetti E Conterno A Greco S Mulatero P Schena M alpha1-adrenergic receptor subtypes in human peripheral blood lymphocytes. Hypertension (1999) 33(2):708–12. 10.1161/01.HYP.33.2.708 10024333

[32] Brodde OE Prywarra A Daul A Anlauf M Bock KD. Correlation between lymphocyte beta 2-adrenoceptor density and mean arterial blood pressure: elevated beta-adrenoceptors in essential hypertension. J Cardiovasc Pharmacol (1984) 6(4):678–82. 10.1097/00005344-198407000-00020 6206325

[33] Feldman RD Lawton WJ McArdle WL. Low sodium diet corrects the defect in lymphocyte beta-adrenergic responsiveness in hypertensive subjects. J Clin Invest (1987) 79(1):290–4. 10.1172/JCI112797 3025262 PMC424047

[34] Swanson MA Lee WT Sanders VM. IFN-gamma production by Th1 cells generated from naive CD4+ T cells exposed to norepinephrine. J Immunol (2001) 166(1):232–40. 10.4049/jimmunol.166.1.232 11123297

[35] Gage JR Fonarow G Hamilton M Widawski M Martinez-Maza O Vredevoe DL. Beta blocker and angiotensin-converting enzyme inhibitor therapy is associated with decreased Th1/Th2 cytokine ratios and inflammatory cytokine production in patients with chronic heart failure. Neuroimmunomodulation (2004) 11(3):173–80. 10.1159/000076766 15067208

[36] Gros R Chorazyczewski J Meek MD Benovic JL Ferguson SS Feldman RD. G-Protein-coupled receptor kinase activity in hypertension: increased vascular and lymphocyte G-protein receptor kinase-2 protein expression. Hypertension (2000) 35(1 Pt 1):38–42. 10.1161/01.HYP.35.1.38 10642272

[37] Iaccarino G Barbato E Cipolletta E De Amicis V Margulies KB Leosco D Elevated myocardial and lymphocyte GRK2 expression and activity in human heart failure. Eur Heart J (2005) 26(17):1752–8. 10.1093/eurheartj/ehi429 16055494

[38] Izzo R Cipolletta E Ciccarelli M Campanile A Santulli G Palumbo G Enhanced GRK2 expression and desensitization of betaAR vasodilatation in hypertensive patients. Clin Transl Sci (2008) 1(3):215–20. 10.1111/j.1752-8062.2008.00050.x 20443852 PMC5350663

[39] Perrino C Schroder JN Lima B Villamizar N Nienaber JJ Milano CA Dynamic regulation of phosphoinositide 3-kinase-gamma activity and beta-adrenergic receptor trafficking in end-stage human heart failure. Circulation (2007) 116(22):2571–9. 10.1161/CIRCULATIONAHA.107.706515 17998459

[40] Gao WQ Han CG Zhao YX Wang Q Zhu P Yang TS [Effect of metoprolol on the expression of GRK2 in lymphocyte of advanced elderly patients with chronic heart failure]. Nan Fang Yi Ke Da Xue Xue Bao (2010) 30(5):1132–3. 20501412

[41] Gao WQ Ma JL Han CG Wang Q Zhu P Yang TS. [Lymphocyte GRK2 expression of the very elderly with chronic heart failure]. Zhongguo Ying Yong Sheng Li Xue Za Zhi (2010) 26(2):207–9. 20684281

[42] Pandalai PK Bulcao CF Merrill WH Akhter SA. Restoration of myocardial beta-adrenergic receptor signaling after left ventricular assist device support. J Thorac Cardiovasc Surg (2006) 131(5):975–80. 10.1016/j.jtcvs.2006.01.027 16678578

[43] Hata JA Williams ML Schroder JN Lima B Keys JR Blaxall BC Lymphocyte levels of GRK2 (betaARK1) mirror changes in the LVAD-supported failing human heart: lower GRK2 associated with improved beta-adrenergic signaling after mechanical unloading. J Card Fail (2006) 12(5):360–8. 10.1016/j.cardfail.2006.02.011 16762799

[44] Akhter SA D’Souza KM Malhotra R Staron ML Valeroso TB Fedson SE Reversal of impaired myocardial beta-adrenergic receptor signaling by continuous-flow left ventricular assist device support. J Heart Lung Transplant (2010) 29(6):603–9. 10.1016/j.healun.2010.01.010 20202864 PMC2876229

[45] Rengo G Galasso G Femminella GD Parisi V Zincarelli C Pagano G Reduction of lymphocyte G protein-coupled receptor kinase-2 (GRK2) after exercise training predicts survival in patients with heart failure. Eur J Prev Cardiol (2014) 21(1):4–11. 10.1177/2047487313491656 23689525

[46] Esposito G Perrino C Schiattarella GG Belardo L di Pietro E Franzone A Induction of mitogen-activated protein kinases is proportional to the amount of pressure overload. Hypertension (2010) 55(1):137–43. 10.1161/HYPERTENSIONAHA.109.135467 19901160

[47] Centonze D Battistini L Maccarrone M. The endocannabinoid system in peripheral lymphocytes as a mirror of neuroinflammatory diseases. Curr Pharm Des (2008) 14(23):2370–2342. 10.2174/138161208785740018 18781987

